# COVID‐19 systematic screening of asymptomatic haematopoietic stem cell donors: Less if often more

**DOI:** 10.1002/jha2.556

**Published:** 2022-09-15

**Authors:** Lucie Blandin, Elise Tolmer, Eric Hermet, Aurélie Ravinet, Amélie Brebion, Richard Lemal, Paul Rouzaire

**Affiliations:** ^1^ Histocompatibility and Immunogenetics Laboratory Clermont‐Ferrand University Hospital Clermont‐Ferrand France; ^2^ Cellular Therapy and Clinical Haematology Department Clermont‐Ferrand University Hospital Clermont‐Ferrand France; ^3^ Virology Laboratory Clermont‐Ferrand University Hospital Clermont‐Ferrand France; ^4^ EA7453 CHELTER Clermont‐Auvergne University Clermont‐Ferrand France

**Keywords:** covid, HSC TRANSPLANTATION, donors

## Abstract

From COVID pandemic spread until now, many HSCT unrelated donor registries recommend as a precaution a systematic COVID‐19 testing for all donors during the precollection time. Literature is quite poor to support this systematic attitude. We report one sibling allogeneic HSCT which we proceeded despite a positive COVID test on related asymptomatic donor and summarize the all seven cases reported until now. We suggest to question this systematic COVID testing, two years after pandemic began, when there is no systematic testing on other blood products received during all the haematological malignancies treatment process.

## 1 INTRODUCTION

COVID‐19 pandemic has disrupted care access for many patients around the world, including those requiring haematopoietic stem cell transplantation (HSCT). Respiratory route was demonstrated as the most common way of transmission of severe acute respiratory syndrome coronavirus 2 (SARS‐CoV‐2) [1]. Blood transmission is largely less documented. Viremia is indeed rarely described in asymptomatic patients tested positive for SARS‐CoV‐2 in nasopharyngeal sample but has been reported in patients presenting respiratory symptoms [2].

Scarcity of the data relating blood transmission had led to great caution during HCST, where recipients are hugely susceptible to infections due to myeloablative conditioning and immunosuppressive treatments.

Systematic nasopharyngeal screening for SARS‐CoV‐2 by real time polymerase chain reaction (RT‐PCR) for peripheral haematopoietic stem cell (HSC) donors before starting mobilization and before donation was introduced in routine practice for some registries. According to these registries guidelines, positivity of one of these samples theoretically contraindicates the HSC collection [3]. In our structure, since the beginning of the pandemic, we have implemented cryopreservation of the stem cell product, to be able to defer conditioning of the recipient, in order to arrange the collection of a back‐up donor if necessary. However, the donation delay and/or the use of an alternative donor can represent a real loss of chance for the recipient.

## 2 CASE REPORT

We report here the case of a 35‐year‐old female diagnosed with acute myeloid leukemia with normal karyotype and NPM1 mutation in 2015. After initial complete remission, she relapsed at molecular level in November 2021. Following a salvage therapy (cytarabine and gemtuzumab ozogamicin), the patient started conditioning regimen to benefit from HSCT with her HLA‐genoidentical sister.

The donor presented a dry cough without any fever or myalgia around the 10th of January but had two negative nasopharyngeal RT‐PCR SARS‐CoV‐2 on the 13th and 15th, and was no longer symptomatic before mobilization. The day before apheresis, the donor was tested positive (RT‐PCR) for SARS‐CoV‐2 but remained asymptomatic, and we thus decided to go through with the stem cells collection on the 20th of January. Before cryopreservation, the graft was tested for SARS‐CoV‐2 by RT‐PCR, which demonstrated the absence of detectable SARS‐CoV‐2. There was no alternative donor and after evaluating the risk‐benefit *ratio*, we proceeded with the transplantation. Neither patient nor donor has been infected by SARS‐CoV‐2 previously, and both received two injections of Pfizer‐BioNTech vaccine.

The conditioning regimen was non‐myeloablative (consisting in fludarabin, busulfan and anti‐thymocyte globulins), started on the 27th of January. Reinjection of the stem cell product took place on the 2nd of February. The patient did not develop any respiratory symptom following the transplant. Neutrophil engraftment occurred at day 18, and the patient was discharged on day 20. Four months after transplant, the patient remains in complete remission, with a mixed chimerism of 98.4% donor and without COVID‐19 infection evocating symptoms.

## 3 DISCUSSION

**FIGURE 1:**
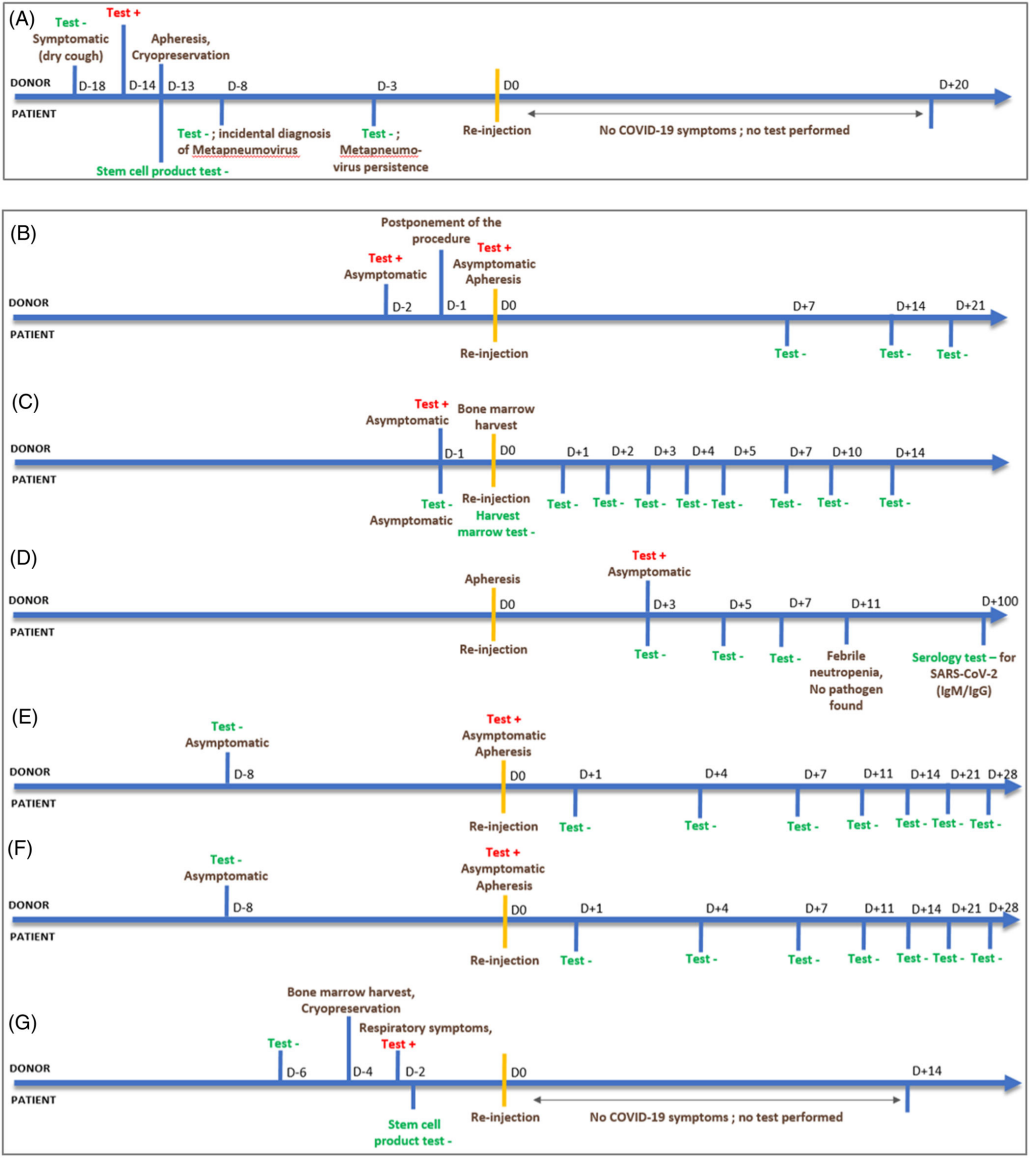
Timelines representing the results of SARS‐CoV‐2 RT‐PCR testing in allo‐HSCT donor and recipients, for the seven reported cases of allo‐HSCT process despite of SARS‐CoV‐2 positivity in donor. (A) Present report (B). [4]; (C) [5]; (D) [6]; (E and F) [7]; (G) [8]. HSCT, haematopoietic stem cell transplantation

More than 2 years after the beginning of the pandemic, it seems legitimate to look back on the practices introduced during HSCT procedures and its consequences. Few other teams have reported clinical cases about patients whose donor was tested positive for SARS‐Cov‐2 but was asymptomatic at the time of collection [4, 5, 6, 7, 8] (Figure 1). None of the recipients was found to have a positive nasopharyngeal test or to develop symptoms of SARS‐CoV‐2 infection following allograft. RT‐PCR tests were performed on only two stem cell products, including ours: both were negative.

Numerous patients have received blood products (whole blood or platelets) from donors found to be positive in the days following donation: no transmission was observed [9]. Even immunocompromised patients did not develop symptoms after receiving blood products from infected donors [10]. These results suggest that the risk of blood transmission of the virus is low.

Furthermore, the requirement of granulocyte‐colony stimulating factor does not seem to have a detrimental effect on the donor. Indeed, all donors with positive nasopharyngeal RT‐PCR were either asymptomatic or developed only mild symptoms, and none of them effectively harvested despite of SARS‐CoV‐2 fortuitously diagnosed had shown any non‐expected complication.

In some countries, HSC donors are not tested for SARS‐CoV‐2 if they are asymptomatic, and cryopreservation is no longer recommended [11]. Indeed, several stem cell products have been cryopreserved but will likely never be infused. In our center, systematic cryopreservation is still the go‐to procedure for HSCT (for both related and unrelated donor). This strategy was chosen to have time to launch the recruitment process of a back‐up donor if the first one is contraindicated tardily.

Our observation, taken together with previously reported cases, raises new questions. With the current vaccination coverage and the very low viremia observed in asymptomatic patients [2], should screening guidelines evolve? Is cryopreservation still relevant for related donors and unrelated donors coming from countries with fast, easy and safe transport of the graft? Also, one can wonder about the impact of a “second choice” donor on the becoming of the transplant: is it better to proceed with the apheresis of the best donor, infected but asymptomatic or to turn to the back‐up donor?

The COVID‐19 pandemic is still here, and we will have to learn to live with it. It is time to use what we learned until now to re‐evaluate our practices and establish worldwide‐harmonized guidelines for recipients and their donors.

## AUTHOR CONTRIBUTIONS

L.B., E.T., R.L. and P.R. contributed to the design of the study. L.B. and E.T. participated in the writing of the paper. L.B., E.T., E.H., A.R., A.B., R.L. and P.R. participated in the performance of the research. R.L and P.R. were involved in critical revision of the manuscript.

## CONFLICT OF INTEREST

The authors declare they have no conflicts of interest.

## FUNDING INFORMATION

The authors received no specific funding for this work.

## ETHIC STATEMENT

This work is integrally included in patient care. Both donor and patient have signed a written consent form relative to the use of biological material and data.

## Data Availability

The data that support the findings of this study are available upon request from the corresponding author.
